# Electrodeposition and Properties of Composite Ni Coatings Modified with Multilayer Graphene Oxide

**DOI:** 10.3390/mi14091747

**Published:** 2023-09-07

**Authors:** Vitaly Tseluikin, Asel Dzhumieva, Andrey Yakovlev, Denis Tikhonov, Alena Tribis, Anastasia Strilets, Marina Lopukhova

**Affiliations:** Engels Technological Institute, Yuri Gagarin State Technical University of Saratov, Polytechnichskaya St., 77, 410054 Saratov, Russia; aselka2796@gmail.com (A.D.); aw_71@mail.ru (A.Y.); tihonov-denis@mail.ru (D.T.); tribis2004@mail.ru (A.T.); buran-92@mail.ru (A.S.); mlopuhova@yandex.ru (M.L.)

**Keywords:** nickel, graphene oxide, microwave radiation, structure, microhardness, corrosion properties

## Abstract

Within the framework of this study, Ni-based composite electrochemical coatings (CECs) modified with multilayer graphene oxide (GO) processed using microwave radiation have been deposited. The process of these coatings’ electrodeposition in the potentiodynamic mode has been studied. The structure of Ni–GO and Ni–GO (MW) CECs has been studied using X-ray phase analysis (XPA) and scanning electron microscopy (SEM).It has been shown that the addition of GO into a nickel deposit contributes to the formation of uniform fine-grained coatings. As a result, the microhardness of the Ni–GO (MW) CECs increases by 1.40 times compared to Ni without GO. The corrosion–electrochemical behavior of nickel CECs in 0.5 M H_2_SO_4_ solution was researched. It was established that the corrosion rate of the nickel–GO (MW) CEC in 3.5% NaCl decreases by about 1.70 times in contrast to unmodified nickel coatings. This effect is due to the absence of agglomeration of the graphene oxide in the volume of the nickel matrix and the impermeability of GO particles to the corrosive environment.

## 1. Introduction

Electrodeposition of nickel coatings is one of the most common methods of protection against corrosion and wear of steel products. Nickel and its alloys are superior to many other metal materials in operational properties, such as resistance to corrosion, wear resistance, and hardness, which results in a wide range of applications (chemical industry, aircraft construction, nuclear plants, medical equipment, etc.) [1,2]. By co-depositing nickel with dispersed particles of various natures, it is possible to achieve a significant improvement in its properties. Metal coatings electrodeposited together with the dispersed phase, which a recalled “composite electrochemical coatings” (CEC), are characterized by excellent physical, mechanical, and corrosion properties.

Thus, nickel-based CECs are known for their excellent adhesion, resistance to corrosion, high strength, and abrasion properties [2]. Among the whole variety of dispersed materials that can be used for obtaining nickel CECs, carbon compounds are of great interest due to their unique properties. Nickel coatings can be modified with carbon nanotubes [3,4,5], graphite [6], nanodiamonds [7,8], fullerene [9,10,11,12], etc.

Graphene and its derivatives occupy a special place among carbon compounds. Graphene is a two-dimensional carbon material having a single atomic structure. Due to the high specific surface area, graphene imparts excellent mechanical properties to the metal matrix compared to all other dispersed materials [13,14,15]. However, graphene-modified coatings are sensitive to surface defects, especially in corrosive environments [16]. The elimination of these and other drawbacks became possible owing to the emergence of graphene oxide (GO), which is an oxidized form of graphene. In graphene oxide, carbon atoms are bonded to oxide functional groups (carbonyl, carboxyl, epoxy, hydroxide, etc.). Because of this, graphene oxide becomes hydrophilic, forms stable dispersions in aqueous and non-aqueous environments, demonstrates high chemical activity, and has electrical resistance [17,18,19]. Graphene and graphene oxide are widely used [20,21,22]. It is known that GO is inert in aggressive environments, which determines the suitability of its use for anticorrosion protection [23]. Thus, nickel-based coatings containing a dispersed phase of graphene oxide can be used to modify parts of micromachines.

It can be assumed that microwave radiation (MW) can change the structure of graphene oxide and improve its properties [19,24]. Therefore, the study of the operational characteristics of the electrochemical coatings modified with GO processed using MW is relevant.

The purpose of this study is to obtain nickel-based composite coatings modified with multilayer graphene oxide processed using microwave radiation in the stationary mode of electrolysis to investigate their structure, physical–mechanical, and corrosion properties.

## 2. Materials and Methods

Composite Ni–GO and Ni–GO (MW) coatings were deposited on a steel electrode (steel 45) from a sulfate–chloride electrolyte bath (Table 1).

Electrochemical deposits of pure nickel were obtained from the above-mentioned bath without a GO dispersed phase (DF).

Multilayer graphene oxide was obtained by galvanostatic oxidation of natural graphite powder GB/T 3518-95 (China) [25] with a current of 200 mA·g^−1^ with 0.7A·h·g^−1^ power supplied to the system in a three-electrode cell with platinum electrodes using a potentiostat P-150x (Elins, Russia).The electrolyte used was 83% H_2_SO_4_, obtained by successive dilution of concentrated sulfuric acid of high purity grade (Russian Standard 14262-78 [26]) with bidistilled water [27].

Microwave processing of graphene oxide was carried out using a ZhUK-2-02 microwave emitter with an operating radiation frequency of 2.45 GHz and a radiated power of 1.3 kW for 15 s.

The phase composition was studied by an ARL X’TRA device (Thermo Scientific, Ecublens, Switzerland) using Cu Kα radiation (λ = 0.15412 nm). The morphology of the dispersed phase and the surface of Ni coatings were studied using an ASPEX Explorer scanning electron microscope (ASPEX, Framingham, MA, USA).

Electrochemical measurements were conducted on a potentiostat P-30J (Elins, Russia). The potentials were set relative to a saturated silver chloride reference electrode and recalculated on the hydrogen electrode.

The Vickers microhardness (HV) of the deposits was measured using a PMT-3 device (AO LOMO, Saint-Petersburg, Russia). A tetrahedral diamond pyramid was pressed into the electrolytic deposits using static indentation under a load of 100 g. The calculation of HV was carried out according to the data of 7 parallel experiments. The error of measurements was 3%.

To evaluate the corrosion–electrochemical behavior of studied coatings, anodic chronovoltammetry curves were obtained in the 0.5 M H_2_SO_4_ (potential sweep rate Vp = 8 mV/s). Corrosion rate studies were carried out in the 3.5% NaCl.

## 3. Results and Discussion

**Figure 1:**
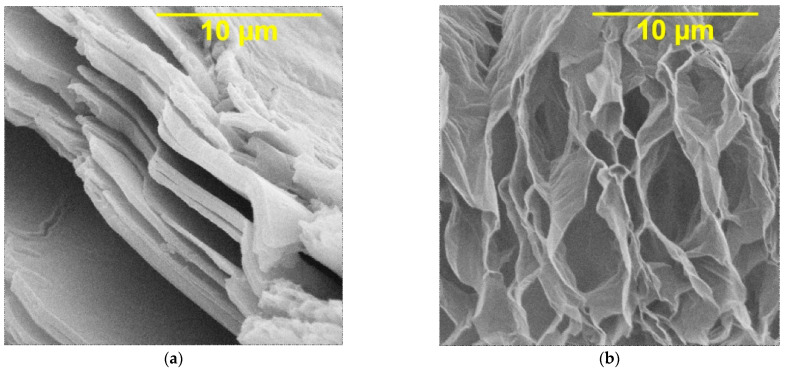
SEM images of (**a**) original graphene oxide; (**b**) graphene oxide processed by microwave (magnification ×10,000).

The properties of composite coatings are largely determined by the properties and structure of DF. Morphological analysis of graphene oxide shows that this compound has a layered ordered structure, the thickness of individual layers being less than 0.1 μm (Figure 1a). Under the influence of microwave waves, the GO layers open, which leads to the formation of a cellular structure with a large number of V-shaped pores with a size of 1–10 μm and the thickness of polygraphene planes up to 0.01 μm and an increase in the specific surface area of graphene oxide up to 400–500 m^2^g^−1^ (Figure 1b).

**Figure 2:**
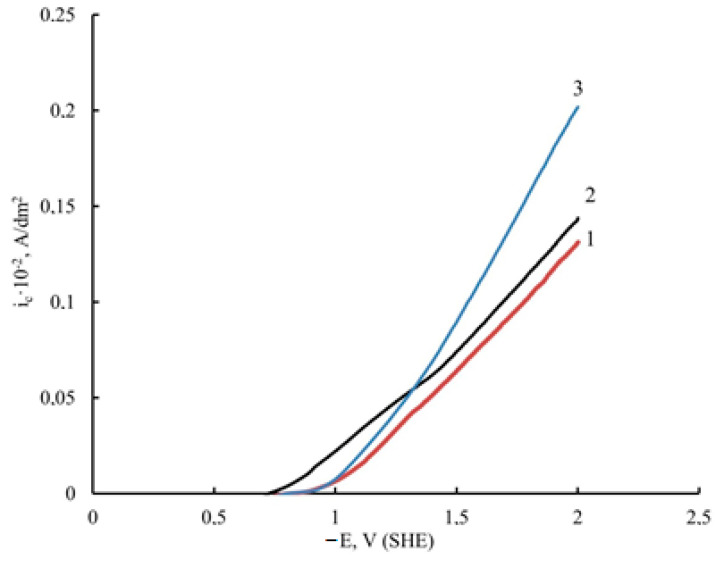
Potentiodynamic polarization curves of electrodeposition of unmodified Ni (1), Ni–GO (2), and Ni–GO (MW) CECs (3) (potential sweep rate Vp = 8 mV/s).

Graphene oxide affects the deposition kinetics of nickel coatings. During the electrodeposition of nickel in the presence of unprocessed GO, the potentials shift to the region of positive values compared to pure nickel (Figure 2); the rate of the process is higher for nickel–GO CEC (Figure 2, curve 2). When the dispersed phase is processed with microwave radiation, the potential shift is smaller than that of the coating modified with untreated DF, but the process rate is much higher (Figure 2, curve 3).

**Figure 3:**
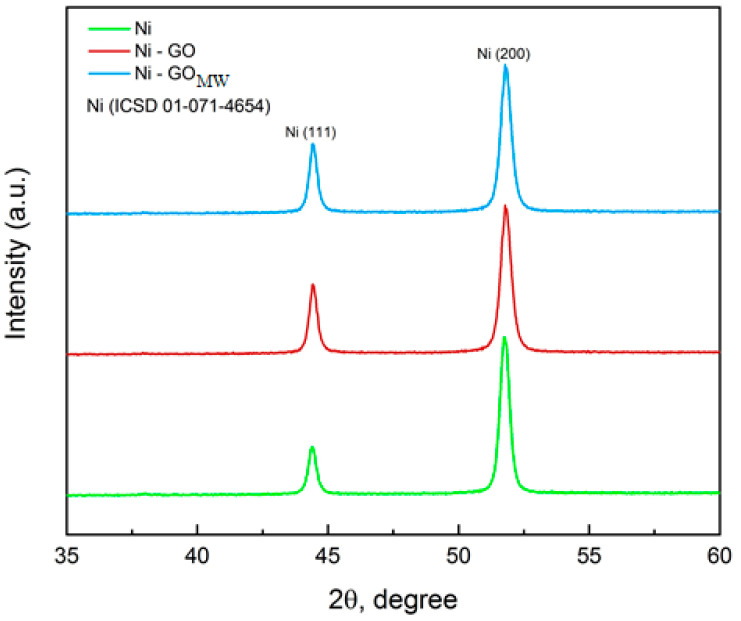
X-ray diffraction patterns of unmodified Ni, Ni–GO, and Ni–GO (MW) CECs obtained at i_c_ = 10 A/dm^2^.

X-ray phase analysis of the obtained coatings shows a change in the crystal structure of nickel under the influence of carbon material. Peaks in the diffraction pattern at 44.5° and 52.2° are responsible for the (111) and (200) nickel crystal planes, respectively [8]. As can be seen from the diffraction pattern (Figure 3), the predominant crystalline orientation for nickel is the direction (200); however, in the presence of graphene oxide, the intensity of the peak at 44.5° increases, which indicates the growth of the nickel crystal also towards the (111) plane. It is obvious that the dispersed phase affects the crystal structure of the nickel matrix. Graphene oxide has a layered structure (Figure 1), and its layers can serve as vacant sites for the growth of metal deposit nuclei, thereby limiting grain growth and making them more compact, which will be further confirmed by SEM images of the studied coatings.

The crystalline size of the obtained coatings at the intensity of the (111) crystalline plane was calculated from the *FWHM* and diffraction angle based on Scherrer’s Equation [28]:

(1) $$D=\frac{K\lambda }{FWHMcos\theta }\frac{180{}^{\circ}}{\pi }$$

where *D* is the average crystallite size, *K* is the Scherrer constant with the value of 0.89 in this experiment, *FWHM* is the full-width half maximum at the peak of 2θ, and λ is the wavelength of Cu-Kα radiation.

The grain size of the studied coatings decreased from 23 nm for the pure nickel to 16 nm for Ni–GO and Ni–GO (MW) CECs. Two types of CEC have the same grain size at the intensity of (111), and it proves our point that there are no changes in the intensity of the (111) plane of nickel modified with microwave-processed graphene oxide compared to the diffraction pattern of the CEC with untreated graphene oxide.

There are no changes in the intensity of the (111) plane of nickel modified with microwave-processed graphene oxide compared to the diffraction pattern of the CEC with untreated graphene oxide. It should be noted that the diffraction peak characteristic of graphene oxide is not observed in the diffraction pattern due to the limited ability of the diffractometer to detect this compound at low concentrations.

**Figure 4:**
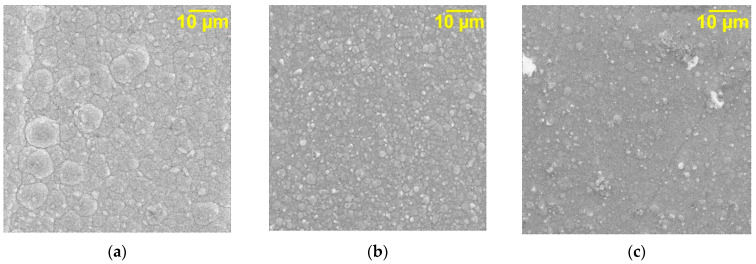
SEM images of unmodified Ni (**a**), Ni–GO (**b**), and Ni–GO (MW) (**c**) CECs obtained at i_c_ = 10 A/dm^2^ (magnification ×2500).

The study of the surface morphology of the obtained coatings shows that the DF affects the structure of the deposits. A pure nickel coating (Figure 4a) is characterized by an X-ray-amorphous, disordered, coarse-grained structure; however, in the presence of GO, the nickel deposit becomes ordered, uniform, and fine-grained (Figure 4b). During the deposition of nickel with graphene oxide processed with microwave radiation, even greater refinement of the grain of the electrolytic deposit occurs (Figure 4c).

Changes in the surface morphology of the coatings and a decrease in the grain size of the deposit directly affect their physical and mechanical properties [7]. Graphene oxide layers in the coating serve as vacant sites for nucleation and prevent an excessive increase in the size of metal grains, which leads to an increase in the nucleation rate. As a result, the number of grains in the deposit increases significantly, as well as the length of the boundaries of these grains, which prevent the movement of dislocations and plastic deformation of the crystal lattice [8]. Moreover, graphene oxide has excellent mechanical properties and a large specific surface area. When composite coatings are subjected to an external load, GO layers can take on some of this load due to the displacement of the shear stress. The motion of dislocations in the matrix is limited by GO layers, which leads to an increase in the energy of lattice distortion and resistance to deformation [22]. These factors ultimately determine an increase in the microhardness of Ni–GO CEC by about 1.20 times compared to Ni without a DF (Table 2). The hardening is caused by a synergistic effect between the formed microstructure of the galvanic coating and the influence of co-deposited graphene oxide. The DF particles themselves also have a strengthening effect on the studied Ni deposits [7,8].

When nickel is modified with graphene oxide processed using microwave radiation, the microhardness of the coatings increases by about 1.40 times compared to pure nickel (Table 2). This may be due to the expansion of the layers of the dispersed phase under the influence of microwave radiation (Figure 2). Expanded GO layers have a large specific surface area, which contributes to an increase in the rate of nucleation during electrodeposition and deceleration of the motion of dislocations in the nickel matrix. Probably, the hardening in this case is described by the Orowan mechanism, according to which dislocations are held on the DF particles during movement until the applied stress becomes sufficient for the dislocation line to bend and pass between them [29,30].

**Figure 5:**
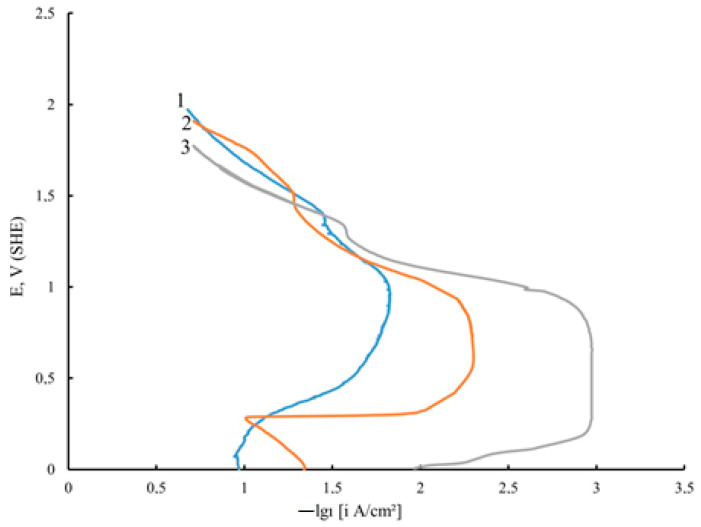
Chronovoltammetry curves of unmodified Ni (1), Ni-GO (2), and Ni-GO (MW) (3) CECs in 0.5 M H_2_SO_4_ (coatings obtained at i_c_ = 10 A/dm^2^).

Another important property of composite coatings is resistance to corrosion. The study of the corrosion–electrochemical behavior of Ni deposits by the chronovotammetry method in 0.5 M H_2_SO_4_ shows that the particles of the DF increase the potential and also reduce the current of the active anodic dissolution of these coatings (Figure 5).

A characteristic feature of the anodic potentiodynamic curves of the Ni–GO CEC is a pronounced passive region, which broadens especially noticeably for the nickel coating modified with GO processed with microwave radiation, while it is significantly smoothed for a pure nickel deposit. Based on the conducted potentiodynamic studies in 0.5 M H_2_SO_4_, it can be assumed that the corrosion resistance of Ni–GO and Ni–GO (MW) CECs will be higher than that of unmodified nickel.

The resistance to corrosion of Ni coatings was estimated from the corrosion rate of the deposits determined by the weight loss of the coatings after they had been kept in 3.5% NaCl for 24 h (the electrodes were weighed before and after they were immersed in a saline solution) using the formula [31,32]:

(2) $$Corrosionrate\;=\;\frac{KW}{ATD}$$

where *K* is constant (8.76∙10^4^), *W* is loss of mass in g, *A* is the working surface area of the electrode (1 cm^2^), *T* is time of immersion in hours, and *D* is density of nickel (8.90 g/cm^3^).

The corrosion rate of the nickel–GO (MW) CEC in 3.5% NaCl decreases by about 1.70 times in contrast to unmodified nickel coatings (Table 3). When analyzing the GO effect on the corrosion rate of the studied coatings, one should take into account the fact that it is due to several reasons. Undoubtedly, the main factor determining the corrosion behavior is the composition and structure of nickel deposits [29]. One of the features of corrosion is its spontaneity, which is indicated by the negative value of the Gibbs free energy of this process. The tendency of Ni to electrochemical corrosion depends on the crystallographic orientation, which determines the surface free energy per unit area of the electrode surface. The ordered fine-grained structure of the studied CECs (Figure 4b,c), in contrast to pure nickel (Figure 4a), contributes to a uniform distribution of the corrosion current over the surface. It should also be noted that the agglomeration of the dispersed phase in the metal matrix does not contribute to an increase in the corrosion resistance of electrolytic deposits and even leads to the deterioration of corrosion properties [33]. Consequently, graphene oxide particles are uniformly distributed in the volume and on the surface of the composite coatings [31,32]. Moreover, the stability and impermeability of the exhaust gas contribute to the lengthening of the diffusion path of the aggressive environment. High impermeability does not allow Ni^2+^ ions to penetrate through the cross-section of the graphene oxide particles [34,35]. Moreover, the effect of the dispersed phase in composite coatings is noted only if the particles form compounds that are more resistant to corrosion than the metal matrix at the phase boundaries or throughout the volume. Obviously, similar compounds are also formed in the structure of the studied Ni CECs. All the above-mentioned factors jointly provide an increase in the resistance to corrosion of nickel–GO and nickel–GO (MW) CECs in comparison to Ni coatings without DF.

## 4. Conclusions

Based on the conducted research, it can be concluded that in the galvanostatic mode of electrolysis, composite coatings are formed from a sulfate–chloride Ni plating electrolytic bath containing a DF of multilayer graphene oxide. The addition of GO particles into the matrix of Ni coatings leads to a change in their surface microstructure. GO has a decisive effect on the physicomechanical and corrosion properties of the investigated CECs, thus contributing to their improvement. Modification of electrochemical nickel with a dispersed phase of GO processed using microwave radiation enhances the positive effect and leads to increased microhardness of the studied coatings by approximately 1.4 times, as well as a decrease in their corrosion rate by 1.7 times.

## Figures and Tables

**Table 1:** Composition of electrolyte and parameters of electrodeposition.

№	Composition of Electrolyte	Concentration, g/L	Parameters of Deposition
1	NiSO_4_·7H_2_O	220	Temperature t = 45 °C
2	NiCl_2_·6H_2_O	40	Constant stirring
3	CH_3_COONa	30	Cathode current density
4	Graphene oxide	10	i_c_ = 7, 8, 9, 10 A/dm^2^

**Table 2:** Microhardness HV_0.10_ values in MPa of Ni-based coatings.

Cathode Current Density i_c_, A/dm^2^	Ni	Ni–GO	Ni–GO (MW)
7	1938	2200	2768
8	2150	2520	2910
9	2350	2938	3223
10	2459	3076	3399

**Table 3:** Corrosion rate, mm/year of Ni-based coatings.

Cathode Current Density i_c_, A/dm^2^	Ni	Ni–GO	Ni–GO (MW)
7	0.656	0.492	0.410
8	0.574	0.410	0.328
9	0.451	0.328	0.287
10	0.328	0.205	0.164

## Data Availability

Not applicable.
